# Elements of the Anatomy of the Human Body in Its Sound State, with Occasional Remarks on Physiology, Pathology, and Surgery

**Published:** 1826-01

**Authors:** 


					V.
Elements of the Anatomy of the Human Body in its sound
State ; with occasional Remarks on Physiology, Pathology'i
and Surgery.
By Alexander Monro, M.L>. F. RlS. ??
Fellow of the Royal College of Physicians, &c. Edinburgh;
Two volumes, 8vo. with Plates. Edinburgh, 1825,
There is no branch of medical science, not even the elegant
and demonstrable discoveries of chemistry, which prolongs
the impression of its own importance to so late an aera of the
physician's life, as his first study, anatomy. It is not that H9
positions are more certain than the truths delivered by other
subdivisions of professional knowledge;' for all truths are
equally certain, though, it may be, not equally palpable, of
equally important; but that the means of verification, of these
truths seldom extend beyond the simple evidence of touch and
sight, and that they form the prime material, and natural founds
tion of all that physiology, and of all that pathology, which, whe'
ther good or bad, every man forms to himself in the direction
his practice. It is from this general predilection for anatomy?
and the sort of average proficiency which it generates in the pro'
fession, that we have always accounted to ourselves for the per'
manency with which good systems of that science so long keep
possession of the schools, and for that rather singular back'
wardness, in a book-making age, of the medico-literary con1'
pilers (so prompt on all other occasions) to supply the defi'
ciencies, which improvement and the incessant cultivation
the science must necessarily leave, at last, in the most perfe^
of systematic works. In short, we opine that, in this in'
stance, the book-makers are, for once at least, afraid of thejf
judges. The announcement, therefore, of a new work of tbi?
kind, always implies greater courage, or more favourable op't
portunities of exercising this " science of mere observation*
than ordinary members of the faculty possess; and it was
1826] j)r . Monro's Elements of Anatomy. 53
conviction that the latter qualification, at least, could not be
wanting in the son and grandson of two of the most eminent
anatomists that Britain has produced, and himself the imme-
11. successor to their museums, their numerous manu-
Cripts, and their anatomical chair, in a crowded and fastidious
diversity?that we were tempted to go through these two
M V .oc*avosj no holiday task to our spectacled eyes, when
r* Weill's dense style of printing is considered. Candour,
9^ver, obliges us to avow that, excepting a few slips of
in 1 WG advertise the Doctor, and the courteous reader
due season, we have not been disappointed in the hopes we
act formed ; but, on the contrary, have gleaned much curious
a original matter from its pages ; and, since our limits will
but of a mere sample of these being introduced, we
a"> for once, let the reader so far into the mystery of re-
e^'lng3> as to inform him that the places of this description
ved *n our notes, and from which the above are selected,
' lount to 90 in number. No rational man expects that any
Systematic work of the present day will ever attain, in the
.??ls, the celebrity or durability of those of the more early
I eriods of science. It might as reasonably be conceited that
modern physician may still arrive at the universal celebrity
I Hippocrates, Paracelsus, Boerhaave, or Sydenham. How
^ort did the fame of Cullen come of all these, though his
ntings are more'"rea(j) and are worth more than those of all
e others put together; and his practice, leaving out Syden-
ani, the only one of the whole that can be reviewed, at pre-
? without commiseration. But Cullen, like the modern
^ystematic writer, found the science in that stage of advance-
Jn^' ^at J^e utmost he could be expected to add or re-model
"e fabric, forms no appreciable object in the optics of the
server, when compared to the stately whole, in the vast pro-
Portions of which it is lost.
hough we cannot then promise to Dr. Monro a more pro-
his^Gd occupancy of the schools than has fallen to the lot of
t , Predecessors, nor flatter him with having far exceeded the
an- \ ^ad ProPosed to himself "of facilitating the study of
lo ?my> Und its collateral and dependant branches,. physio-
cuP'i Pathol?gy5 and surgery," yet we say that he has exe-
n ? tas^ *n a manner not unworthy of the illustrious
^me he inherits, and that he has rendered his attempt to do
ni hly interesting by a great mass of curious and original
, a r> derived from sources accessible only to himself, or his
o m.erous scientific friends, whose talents indeed he appears
au occasions to have willingly directed towards the eluci-
to
II
54 Medico-chirurgical Review. [January
elation of the obscurer parts of his favourite science. Pr*
Thomas Thomson, Dr. John Davy, Dr. Brewster, Mr-
Bauer, Sir Astley Cooper, Professor Hamilton, Dr.
Knox, Dr. Holland, Sir Andrew Halliday, and a mul-
titude of other scientific gentlemen, have all been laid under
contribution for the present work. Of course, the variety and
interest of their communications are very great; but might, i'1
any other work, rather have tended to distract than engage
the attention of the juvenile reader, for whose assistance the
work is mainly intended. But Dr. Monro has got over this
difficulty with more dexterity than might have been expected,
in a mere work of anatomy. This he has accomplished,
partly by judicious division of his subject under proper heads,
partly by pursuing the method he follows in his lectures,
presenting the student with matter naturally miscellaneous,
under the designation of " General Observations " upon the
individual sections of his science. We have always though1
this method advantageous, as it both renders the substance
of the sections themselves less rickety, and liberates the
teacher from the trammels of method. We find that we dis-
like it as little in books, and, in fact, are inclined to impute
the felicity of our author, in this particular, chiefly to its pro-
per application. It is time, however, to introduce our reader
more analytically to the work itself, that he may be the better
enabled to compare our opinion with his own judgment. The
order and division observed in the arrangement of the book is
as follows.
J Volume I.
I. The Bones and Periosteal Tissues.
II. Cartilage and Articulating Tissues.
III. Muscular Tissues.
IV. Digestive Apparatus.
Volume II.
V. Circulatory and Respiratory Apparatus,
VI. Glandular and Urinary Apparatus.
VII. Generative Apparatus.
VIII. Vascular System.
IX. Brain and Nervous System,
X. Apparatus of Sense.
XI. Lymphatic System.
XII. Apparatus of Evolution,
Though we account it nought to be angry, in limine, on
small a matter as arrangement in anatomy, the science whi^1
J
1S26] j)r, Monro' a- Elements of Anatomy. 55
of all others, must, after every effort, still most egregiously
anticipate, yet we find serious fault with Dr. Monro for hav-
lng interposed the digestive apparatus between the muscular i
and arterial system. We know he will say that the apparatus
m question is muscular, and that he also considers the vascular
system as such in a lesser degree, and, therefore, entitled to
a place after it; but this will never do. Neither the one nor
the other is of the least moment as a muscular tissue ; they
both derive their name and their importance, not from their
texture, but from their relation to the existence and support
whole economy; and from this, also, should they derive
their location in a regular system of anatomy. We were
shocked, also, to find nearly 100 pages of excellent, novel,
and original matter, comprehending the distribution of the
nervous system, thrust in at the end of the section on the skin,
??% 100 P ages from its natural situation, which is at page
a.85> or the termination of the history of the brain. Was it.
that Dr. Monro, during the printing of this part, became
aware, through the journals, of the great discoveries going for-
ward, and was willing, at the sacrifice of a little strict method,
t? receive them into his pages in a more perfect form, though
a less suitable position of his arrangement ? We are quite
ready to give ourselves credit for the sagacity of this conjec- j
Ure; else we should be but ill disposed to pardon that osci-
dnt appearance which such a dreamy lack-rule disposition
n*ust give to the most valuable materials. With these excep-
ts, the arrangement is respectable.
. Ihe names of Monro and Osteology are so early associated
]n the mind of every medical man, that we almost instinc-
uvely turned to that part of the work, as a specimen of what
^ght be expected from its other sections, and were indeed
delighted witli the manner in which it is executed. We know
11(Jt whether it has been remarked that, with all the native ex-
lence of the first Monro's work on this subject, and the
valuahle alterations that have been wrought 011 it by various
editors, that it is still very deficient in proper appellations to
niany parts of the bones which it describes with graphic
accuracy, but which, in that author's time, had not received
distinct names. The descriptive sciences were yet in their
infancy; no Linnajus had appeared, to give a system and a
language which might be easily accommodated to all the 111-
varieties of form and existence which Nature exhibits in
he created world; and ignorance and youth, which even to-
1 are but slowly reconciled to the utility of nomenclature,
Would, in those early times, have revolted at a more prolix
56 Medico-chirurgical Review. [January
array of names, even from the masterly hand by which they were
conscious it was offered. Now, however, that matters in this
respect are much improved, our author has done right to adopt
the language and nomenclature of his ancestor to the present
time, the beautiful, almost poetic exactness of that great
man's description, leaving it hopeless to his grandson, or, in-
deed, to any one else, to supplant it by an original production-
The great additions made by our author, however, even to the
descriptive parts, the numerous sensible pathological and prac-
tical observations every where subjoined, the distinct original
chapters and entire articles interspersed, sometimes from his
own observation, and sometimes from the unpublished manu-
scripts of his father and grandfather, render the osteology o>
the first volume of this work the most interesting and instruc-
tive of any with which we are acquainted in our language-
The perpetual applications of his observations to the practice
of medicine and surgery, must be highly edifying to the
younger part of the profession, who, as Cheselden justly re-
marked, always " require the most efficient information with
the subject under their noseand the curious, though more
sparingly added references to zoology, cannot fail to delight
the anatomist, and student of nature. The author seems to
? have profited by the method pursued by Treveranus in his ad-
mirable "Biologie;" at least we find him referred to in the
book, and the principle is the same. But we hasten to flou-
rish the shears?not of Clotho, indeed, for these we reserve for
our patients; but of the gay, versatile Vertumnus whose vo-
taries, be it known, in their transitions
" From grave to gay, from lively to severe,"
are often more indebted to that humble instrument, than the?r
own wit, for the amusement or instruction afforded to theu
readers. The following is the Doctor's account of the for''
mation of bone, p. 47. In it, he appears to have decided thc
question of red effusion during the formation of bone.
Dr. M'Donald's experiments led him to suppose, that pure bloolj
is effused between the broken ends of the bones, from which the re
globules are, in a short time, extracted.
*' This coagulated blood, or incipient callus, in a short time, he'
comes vascular, and is covered by a newly-formed periosteum, wbic
adheres to the original one, after which the ossification commence*
But the breaking the bones at the commencement of the experime'1''
by which many blood-vessels may have burst, seemed to me to render
it impossible to determine whether the effusion of red blood was t'j"
preliminary step. I, therefore, directed my attention to that remarks!'e
disorder, the hydrocephalus chronicus, in which the bones of the sku"'
A
1826] j)r Monro's Elements of Anatomy. 57
ln consequence of the increased volume of the brain, are kept crowing ?
t<a "uxnber of years.
i. * ? consequence of having examined a number of instances in
'ch bone was forming, I am led to believe, that the process of the
?rmat'?n of bones, is as follows :
. "lood is effused, the red particles of which are speedily absorbed,
ge her with the serum. The fibrin which remains, forms a sort of
tafc'US' 1D Which l'10 bony matter 's deposited; whilst this process is
Tti^h ^ace> blood-vessels of the part are very much enlarged,
in 61 . y matter is not deposited in a solid form ; it is probably held
bv f? on the phosphoric acid, which acid is afterwards abstracted
re l!j absorbent vessels. When the bony substance which has been
nt'y formed has been dried, it is very white, and so soft that it
^vi.- , scratched by the nail, and exhibits a granular surface, but
the a^8r a sbort time, it loses (in consequence of the filling up of
^/oterstitial spaces) and becomes quite smooth. It merits particular
that these small grains, which constitute as it were the bases of
?nes, are not always of the same size.1'
Cpi^e blowing remark, as it respects the remains of the
curated hero and liberator of Scotland, Robert I. of that
u ,,?m> maY be worth a place.
in f bones when dried do not change their figure ; and do not,
da/0?16 circumstances, for a great length of time, crumble into
, ^o illustrate this fact, it may not be improper to mention, that I
de*^ nCcas'on> (^th November, 1819) to examine the skeleton of Robert
^as /Uce> who died A. D. 1350. On opening the vault, the body
VVrS tound included in thin lead (which was partially oxidated) and
apt up in an embroidered linen cloth. All the softer parts had dis-
appeared, but the skeleton was perfect, excepting a few of the smaller
Co|Qes the feet. The bones were somewhat moist, of an orange
bit Ur' an^ was remarkable, that even the very thin bones of the or-
of ^Gfe clu^te entire, as also all the processes of the bones, at the base
, e skull even the pterygoid processes of the sphenoid bone, and
the Palate bones."
au^lor afterwards, table ii. p. 203, relates, that this
UU was of uncommon dimensions, being twenty-two inches
the a (luar^er ln circumference, seven and three-quarters from
fro n?Se 1:0 os occipitis, and three and seven-eighths across
ag m the origin of the coronal suture. We take the following
an example of avast number of other similar ethuographic
Servations scattered over these two volumes.
The thickness of the bones would seem to depend on the nourish-"
Hence, the natives of Australasia and Van Dieman's Land,
? Were ignorant as to the means of raising the necessary quantity
n?Urishment, were tall, but very slender. My friend Sir John
58 Medico-ciiirurgical Review. [January
Jamison sent me, some years ago, the skeleton of a young man, a fla'
tive of New South Wales. The bones are rather smaller than thoge
of the females of the higher orders in this country; the chest and pe'"
vis are very narrow."
Respecting the induration, or actual aggregation of bo?e>
Dr. Monro makes the ensuing appropriate remarks.
" But the above theory of my grandfather, Baron Ilaller, and othetf?
is inadequate to the explanation of the process of ossification, which,l0
many cases, takes place where pressure cannot exert its influence.
" Bony matter is secreted from the blood like the bile or urine, aO<l
formed into its proper consistence by the action of the circulating an^
absorbent vessels, independently of pressure, which cannot, therefor6'
be admitted to be the principal cause of ossification. Pressure, after
the bones have been formed, may, perhaps, have some effect in coD'
densing them.
" Climate may also accelerate the process of ossification; whence
in hot climates, the inhabitants sooner arrive at their full stature tha"
in northerly cold regions."
This curious question of the aggregation of bone is certain'/
not unimportant; and our author's observations are accural
and sensible as far as they go. But the thing is not so
ob'
scure as our wild theories have hitherto led us- to suppose, aJlC
the difficulty has all lain in the unwillingness of the two sect*
] of Mechanists and Fi talis ts, into which medical men have iM
divided themselves, to concede any point or portion of an agret"
ment that might seem to favour their adversaries. Look
a stalactite?see it issue from the bowels of some rocky cavefl'j
a simple solution of chalk in an excess of carbonic acid, a11
clear and transparent as the water which supports them both*
The excess of acid, which was only retained by pressure, a'1
strongly attracted by the aqueous vapour and gaseous element
of the atmosphere itself, must needs escape as soon as it comel3
in contact with the latter; the carbonate of lime is, therefo^
\ precipitated, and in what form? A rude, shapeless jelly, ?f
which the corioid figure is determined by the most simple
all circumstances, its own gravitation to the centre. But e*'
amine^the calcareous icicle so produced some months aft^r'
wards, and you find the external surface, indeed, covered w^1
the same weeping semipellucid jelly as before, but the centre^
a mass of distinct regular crystallization. You have here tftej
a case almost exactly similar to the consolidation of bone, ai'jl
precisely like that of the crystallization of urinary calcul1
within the bladder. The two latter, say the vitalists, deri^
their hardness and rectilineal structure from the operations
the principle of life: but where, we would ask him, is the v1'
1826]
Dr. Monro's Elements of Anatomy. 59
1
tol spirit which consolidates the lonely stalactites that beautify
sparry caverns of Kentucky and Indiana ? That vital prin-
ciple we doubt not is common to both, as it is to all nature,
^eing the mere attraction of aggregation, modified by the gra-
dual removal of the water which was originally interposed be-
^yeen them. In the stalactite, this is effected by the three <4-..
gradual processes of evaporation, hydration, capillary attrac-
tl?n: in the bone, by vascular absorption, by hydration, (for
*10 unliydrated salt has ever been discovered in the animal
"?dy) and, if Magendie is right, by imbibition into the sur-
rounding less humified tissues: and thus, that concentration of
fluids, which nobody wonders at producing crystals in cliemis-
^ry, because it is the common process for effecting this conso-
lation, operates this curious induration simply by bringing
jhe molecules within the sphere of each other's action. Finally,
the stalactite be placed in the situation of the bone, and it
^vdl no doubt have its rate of consolidation modified by the vi-
tal activity of the absorbents, its form by the surrounding parts,
a,1d the vigour of deposition ; but the adhesion of its particles
each other will ultimately depend upon the great law of at-
l^ction. This is our view of the aggregation of all animal so-
Ms, which constantly approaches that of inorganic nature, in
Proportion as the quantity of insoluble matter is increased, ^
?nd the quantity of solvent diminished, circumstances which,
ltl a living body, are greatly modified by the operation of living
agents; and, in the operations of brute matter, by the agency
atmospheric changes, which constitute the stirring prin-
ce, the cause of increase, change, decay, and reproduction,
^ the inanimate world. It is not impossible that, from
same view, an explanation of rickets, malacosteon, fra-
SlUtas ossium, exostosis, and other diseases, might be easily
derived.
The following careful measurement of the pelvis is valuable.
, ' The axis of the pelvis varies much in the different attitudes of the
b?dy, and even in the same attitude at different periods of life.
, " In the erect position, the line of the axis of the upper aperture of
pelvis, extending between the navel and the extremity of the ossa
c?ccygiS) forms a considerable angle with the line of gravity of the body;
and hence the bowels of the pelvis are well supported: owing, also, to
le oblique position of the pelvis in the erect posture, and from thepe-
Culiar position of the thigh bones in respect to it, the line of support cor-
responds with that of the lower extremities.
, " The axis of the inferior aperture, is nearly in a line with that of .
e vagina, and forms a considerable angle with that of the upper ape-
Ure: labour, therefore, it more difficult in the human race than in in-
?i?r animals.
00 Medico-chirurgical Review. [January
** The pelvis varies much as to its size, and the proportions of i'3
different parts in different women, in the inhabitants of different nation^
and also at different periods of life.
" In the standard pelvis, a line drawn from the sacrum to the pubi*?
measures rather more than 4 inches, from one os ilium to the other/
inches and |; and in the lower part, or inferior aperture of the pelvM
these proportions are reversed ; for a line from the symphysis pubis,
the os coccygis, allowing an inch for the retraction of that bone, is eq"3'
to 5 inches. The standard pelvis is about 3| inches from the brim t0
the tuberosity of the os ischium, G inches behind, and the depth of
symphysis pubis is about \\ inch. ,
" In order to throw light on the measurements of the female ^
male pelvis, I selected several pelves of both sexes, which I consider^
to be very well formed, and also several that are very much deformed >
and I have subjoined a tabular view of the measurements, taken in
ferent- directions, by my excellent assistant Mr. Mackenzie. All
measurements were taken from inside to inside.*
44 In the subjoined Table, I have given the measurements only of ^
pelvis of one Negress, as the other I possess, which was also sent to
by Professor Horner of Philadelphia, corresponds, in all respec's
with it.
44 It may be proper also to state, that the pelvis of a Negro, whi^
the same gentleman sent to me, is rather less than that in the Table.
^
" * In my Outlines of Anatomy, I published many valuable observation'
on .the pelves of women of different nations, by that justly celebrated an^0'
mist Dr. P. Camper, which are illustrated by a diagram drawn by himse"'
to which I refer the reader.
" Mr. Mackenzie, from his zeal to communicate information, meas?re '
after the types of this sheet were set up, five female pelves, in the p?sSf,
sion of a friend, which he found to measure somewhat less than those of ^
subjoined Table.
Dr. Hull's tables as to the distorted pelvis merit the particular att?"'
tion of the reader."
?' I
A
1826] Z>r. Monro's Elements of Anatomy. 61
" Tablk I.?Measurements of well formed Pelves.
)
2
3
4
5
6
7
8
9
10
H
Egress,
Male
Negro,
Male,
Australa-
sian.
EC
Z a.
? H
? >?
cS CO
s s
65
I!
*s o
4 1
IT
44
44
44
44
4-;
34
4J
4*
T
S-3
? S
E ?'
o*. ?
iT &> 3
?o|
5f<
3-S 4>
c*b?.c .
~ E " ?
?
Oto o 3
44
41
%
43
*5*
IT
?T
5|
51
44
41
4
4i
54
44-
s?
E ^
o a
c
5 S
p< 5
5 ?
41
%
3f
44
34
44
34
? .
> .-
C O
4a
IT
5t
34
34
3f
3t
K-~
E~
o
? s
0) >?
(J CO
? v
*?2
5
44
T
54
5r
44
31
? Q-
* E
? r*
4?
46
%
41
4f
4 3
%
44
II
ts
?s
c u ?
rt aJ .-
5i
54
54
*?1
0T
06
"IT
44
4f
/*. in.
5 2
5 4
4 6-j
5 6
5 6
6 0
5 6^
Table II.?Measurements of Deformed Female Pelves.
14
T
34
Right 4
Left 4^-
Right 3|
Left 3
4|r
44
4i
4f
4|
24
44
34
H
Left 3i
Right 3
4i
4
G2 Mkdico-chirurgical Review. [January
" From the table it follows,
" ls?, That in a well formed pelvis, the antero-posterior diameter is
sometimes equal to four and a half inches ; but that in the Negress, it is
bonsiderahly less ; and, in the female deformed pelvis, it is sometimes
equal only to one and a half inch : That in the male, it is not propor-
tioned to the stature of the body, for in the skeleton which measured
six feet, it was less than in the other two male skeletons of inferior sta-
ture.
" 2d, That, in a well formed female, the conjugate diameter of the
pelvis generally equals five inches, and sometimes exceeds it a little;
but in the Negress it is less, and is also still less in three of the deformed
pelves. The mollitie3 ossium, therefore, does not tend to diminish this
diameter of the pelvis so much as the antero-posterior diameter.
" The oblique diameter of the malejpelvis did not (excepting in No.
10.) equal that of the female pelvis.
3d, That the lateral diameter of the pelvis in the skeleton is greater
than the oblique ; but in the recent body, it must be less, this aperture
being diminished by the psoEe and iliaci interni muscles; and it merits
notice, that the mollities ossium does not materially lessen the lateral di"
ameter of the upper aperture of the pelvis.
" This diameter is not so great even in a tall man, as in a female who
is shorter by several inches.
" 4th, That the depth of the os sacrum, from top to bottom, was less
in the Negress than in the European, and still more so in one of the
deformed pelves 1 measured ; and in the other deformed pelves, it was
reduced even in a greater degree.
" In the Negro, also, the depth of the os sacrum was inferior to that
of the sacrum of the male European.
" 5th, That the transverse diameter of the outlet varied considera-
bly, even in the well-formed pelvis of the European female, in some
specimens exceeding 5 inches; but in others exceeding by a little 4
inches only : that in the Negress, it was nearly an inch less than in the
European, and in one of the deformed pelves it equalled only two
inches.
In the Negro and male European, there was but little difference as to
this diameter of the pelvis, but it was rather less in the male Australasia^
than in the male European.
" 6th, That the distance between the extremity of the os sacrum and
the symphysis pubis was less in the Negress than in the female Euro'
pean, and rather less in the Negro than in the male European. In
three of the deformed pelves, the distance equalled little more than 3
inches.
" 7th, That the distance between the sacro-iliac symphysis, and sym'
physis pubis, was also less in the Negress, and in the male from Austra-
lasia, than in the European female ; and still less in one of the deformed
pelves.
" 8th, That the distance between the fourth piece of the os sacrum
and under part of the symphysis pubis, was much less in the Negres3
A
1826] Z)r. Monro's Elements of Anatomy. 63
fg mPared with the other diameters of the pelvis, than in the European
in i ' an^ ^lat t'1's diameter in the deformed pelvis was also less than
^ e Well formed pelvis.
tlia short, the pelvis of the Negress is smaller in all its diameters
tc^H ^le ^ema^e European ; and the same holds true in respect
tt10 Negr?, when compared with that of the male European.^
^ he preceding table also indicates the distinctive characters of the
"a?f ^emale pelvis.
?he b ? ^ema'e pelvis is evidently larger in all its dimensions, except
c.;tl- "e,ght, than that of the male: hence women take more room in
S|tting.
ter ^heossa ilia spread out wider, and thus the enlarged womb is bet-
sor SUfP0rted. The brim or upper aperture of the female pelvis is
cliil j at an oval shape, corresponding in form to the head of the
'? ?^the roale more nearly approaches to a circle.
e<*ch directing the attention to the several parts of the female pelvis
ence 13 ^?Un^ to be proportionally larger, so that the greater circumfer-
oj.e?fthe female pelvis does not depend upon the greater expansion
Cocac?y?ne particular part. The incurvation of the os sacrum and ossa
Us i^1S' wh'ch is peculiar to the human race, is greater in the female,
sev S<i breadth of the bones; and the bond of union between the
*heseh '0mPonent pieces of the oscoccygis is more loose, which renders
L bonesmore moveable ; and hence they may be pushed backwards
Un ], an i"0^ during labour, which considerably enlarges the
: ^^perture of the pelvis, and facilitates delivery.
fcfF s*ze *he notc^ i? the ilium is larger in the female : the
r1LCt of this is, that, by encreasing the arch, the ossa ischia are at a
cirr er d'stance from the os sacrum, and from each other; from this
u ^ftance? therefore, the breadth of the pelvis is greatly increased,
ly he greater size of the foramina thyroidea has not been suflicient-
tircleGn l? * *^us ^le conj?ine<^ os ischium and pubis form a larger
" Tl
in ^ le upper aperture is also considerably larger in the female than
?hat h Ina^e pelvis, the os sacrum is broader ; and the distance between
there "?ne anc^ ^le symphysis pubis is greater ; and in the recent body,
tartiia1S 3 remar^a':,le difference in the thickness of the one or the two
mal a#es between the ossa pubis, which are not so deep as in the
an The ossa pubis in the male, form, by their conjunction,
Jn ?LCUte angle; but, in the female, the angle is wider considerably.
ar>d cavity ?he pelvis, or true pelvis, is larger in the female,
hr0a", Ca.rs a greater proportion to the upper part of the pelvis. It is
the er 10 its dimensions ; hence the tuberosities of the ossa ischii,
fjQ^^hula, and spinous processes of the ossa ilii, are farther distant
tjip. ea.ch other ; and of course the thigh-bones of the female are placed
^obliquely." 85.
^ide Tenon, Mem. de l'lnstitut. des Sciences, torn. vi. p. 1/2.
64 Medico-chirurgical Review. [January
We regret that our limits will not permit us to insert the
original essay on the Skulls of different Nations, nor the ar-
ticle upon the frontal sinus, and the proportions of human
beauty ; and extracts would convey to the reader no proper idea
of the tracts themselves, to which, therefore we are content to
refer the reader. The chapter, (ix. p. 182) " of the causes
which determine the size and form of the skull, and of those
causes which lead to an alteration in its shape," also overflows
with curious matter, which cannot be extracted. The next
" of the distinctions of the skull of the male and female, and
of the distinction of the different nations," is a necessary and
proper part of osteology, though here, almost for the first time?
introduced into a regular system of anatomy. It was formerly
studied, however imperfectly, under the head of natural history*
but is purely anatomical. The doctor has collected from every
source, has explored his own extensive collection, and enriched
the whole with numerous tables, and an account of the simple
craniometer, (p. 202,) by which he finds that the cranium, that
most untractably irregular cumulus of protuberances, " may be
transferred to a piece of paper, copied or reduced with mathe-
matical accuracy, and every deviation from the most common
shape, may be traced and represented on paper by one who has
attained but little proficiency as a draughtsman." What ai'c
the persecuted proselytizing phrenologists about? A single
dozen of living craniums taken at random in a pothouse, and
laid faithfully down in this manner, would do more to establish
their doctrine than all the volumes that ever were or will be
written?provided its squares told no tales. Crania, th?s
traced, may be seen in plate xx. of our author's outlines. Tbe
artifice merely consists in placing the cranium within the are*1
of a moveable parallelogram, between whose opposite sides a
number of silk threads, crossing at equal distances, divide tbe
cranial area in view into equal spaces.
In myology, our author is chiefly distinguished by his n0'
menclature, the principle of which is to frame the names o*
these numerous organs upon the place of their origin and insef
tion. The objection which has been made to this, is that man)'
muscles have more than one origin or insertion, and thus swe"
the name out to a most unpronounceable length, while of other3
again, it is difficult to say which part is the origin and whi^1
the termination. Now there is not much in all this ; in tb?
abstract, every name should convey some idea of the thin#
named, and that there is something revolting in the presenj
nomenclature of muscles is proved by the difficulty which
feel in recollecting them, and the rapidity with which the bnHv
1826] Z)r. Monro's Elements of Anatomy. ^
?f medical men forget them, though their number does not
greatly exceed that of the bones, which are never forgotten.
^r. Monro has certainly not been successful in removing this
jeproach from myology; but his attempt deserves credit, both
because he is in the right path, and because he is not deterred
by the coldness and opposition with which it is received by the
learned. We have a suspicion that something might be done
"y dint of mere grammar. Why, instead of such words as
atloido, suboccipitalis, or praedorso atloides, which we take at
mere random, and which prefigurate (it may be necessary to
inform the reader) the rectus capitis lateralis and longus colli,
may he not say occipatlas and vertebratlas, sounds little re-
commended by their euphony, to be sure, but not exceeding
the quadrisyllable average of our scientific terms, and com-
pounded according to the genius of the learned tongues from
AvJiich they are derived. It is nothing that some muscles have
m?re than one origin and insertion. Let Dr. Monro always
8.elect the chief one alone, let him avoid descriptive designa-
tions, and abbreviate the root-names in a manner corresponding
to the rules of grammar, and we shall not hesitate to foretel a ,
more favourable reception to his nomenclature.
At page 531, we have some interesting experiments of the
author's on the section of the 8th pair and great sympathetic,
which are perfectly in harmony with those of Magendie. At
57 we find some ingenious criticism on the colours of organs,
^9 which Dr. Monro has paid the most praiseworthy attention.
Npt a day passes over our heads, in which we are not shocked
with some absurdity in the description of dissections, which is
entirely attributable to neglect of this circumstance. This
v?lunie concludes with a superior original article on biliary
Ctilculi, by Dr. John Davy. r 0
In the second volume, to follow the Professor through his
observations on paracentesis, the muscularity of arteries, for
which he is a zealous stickler, the doctrine of asthma being a
luryngeul disease, his claim to the valuable stomach pump, -
lately brought into use by Mr. Jukes, his excellent precepts for
m.ssecting the perineum, his admirable description and engra-
^ng of the fibres of the bladder, his doctrine of the tissue of
the penis, so totally opposed to Magendie, in his second edition,
..ls respectable articles on the urethra and practical remarks on
*t 'iotomy ? on the occasional or intermittent loss of sight, on
tensor tarsi horneri, on the musical ear,* and a multitude ? ?,
?; 7
^Ve were pleased to find the Author agree with us in lauding the meri-
r,?.Us acoustic labours of Mr. Buchanan, of Hull.
V?>~ IV. No. 7. F
I
I
k?
I
$
u
66 Mbdico-chirurgical Review. [January
of others, would tire both writer and reader, hut may safely be
recommended by the former to the latter. It is our part to
point out what we think valuable to the "kenner," as our
German cousins denominate a connoisseur, and to bear our
faithful testimony to the probable usefulness and certain ability
to be found in the perusal of the book. We regret exceedingly
that the respectable publishers and printer allowed it to go into
the world so wretchedly corrected, (we speak technically,) as it
at present stands. Nor can we entirely exonerate its author
from all blame in this respect; for though the artists are most
to be found fault with, yet no author, however high his charac-
ter, or respectable his station in the profession, ought to think
himself above the humble task of revision; however great he
may be, the public which he addresses is still greater. We
have ourselves woeful experience of the impossibility of attain-
ing perfection in this respect, and can make every allowance
for the simple reliance on others, or even the weariness of an
author in the printing of a long work ; but as to us, an Edin-
burgh professor is, in divinity phrase, only a mere man. We
must say a weighty fault exists somewhere, and that we desi-
derate, both proper attention and care in the correction, a
proper alphabetical index, and a due regard to order in the dis-
posal of the contents of the book. Whoever can dispense with,
or pardon these, will find he has made a reasonable purchase
of curious and recondite anatomical information mixed up with
the elementary science of that important study.

				

